# Circumscription and Taxonomic Arrangement of *Nigroboletus roseonigrescens* Gen. Et Sp. Nov., a New Member of Boletaceae from Tropical South–Eastern China

**DOI:** 10.1371/journal.pone.0134295

**Published:** 2015-08-11

**Authors:** Matteo Gelardi, Alfredo Vizzini, Enrico Ercole, Egon Horak, Zhang Ming, Tai–Hui Li

**Affiliations:** 1 Via Angelo Custode 4A, I-00061 Anguillara Sabazia, RM, Italy; 2 Department of Life Sciences and Systems Biology, University of Turin, Viale P.A. Mattioli 25, I-10125 Torino, Italy; 3 Mikrobiologisches Institut-Universität Technikerstrasse 25, 6 Stockwerk, A-6020 Innsbruck, Austria; 4 School of Bioscience & Bioengineering, South China University of Technology, 510006 Guangzhou, China; 5 State Key Laboratory of Applied Microbiology (Southern China), Guangdong Institute of Microbiology, 510070 Guangzhou, China; Field Museum of Natural History, UNITED STATES

## Abstract

*Nigroboletus* is proposed as a novel genus in family Boletaceae, subfamily Boletoideae, to include *N*. *roseonigrescens*, a new boletoid species from tropical environment in south–eastern China. Detailed morphological description, color pictures of both fresh basidiomes in habitat and dried material along with photomicrographs and line drawings of the main anatomical features are provided, supported by a comprehensive phylogeny based on multigene molecular analysis (nrITS, nrLSU, *rpb1*, *rpb2* and *tef1-α* datasets). Taxonomic placement and evolutionary relationships of *Nigroboletus* are investigated.

## Introduction

In recent times Chinese bolete diversity has turned out to be quite impressive and much richer than formerly thought. As a result, several unnamed genera and species belonging to the order Boletales have been documented as new to science over the past decade, based on bio–geographic evidence and mostly supported by multi–locus molecular phylogenetic inferences [1–23]. However, as outlined by a number of recent publications, boletes heritage in China is currently far from being fully understood and a large amount of fungal taxa are still waiting to be uncovered or formally recognized [24–26]. As a matter of fact it is relatively easy to find out boletoid species which have been previously misidentified or even completely overlooked, especially in tropical or subtropical climates where fungi are underdocumented and mycological research has significantly been less extensive than in northern temperate and boreal regions [27–29]. Based on recent sampling from tropical south–eastern China (Guangdong province), a new member of family Boletaceae, subfamily Boletoideae ([26]; corresponding to the “anaxoboletus” group in Nuhn et al. [30]), has been recovered and carefully examined; the new taxon is promptly circumscribed by a combination of macro—and micromorphological features including the medium–small habit of the basidiomes, the pastel rose pileus, the tissues turning blackish when injured, the small, broadly ellipsoid to subovoid spores and the erect subparallel to loosely interwoven pileipellis structure.

According to morphological characters and multilocus molecular inference, a newly described genus is necessary to accommodate the undescribed taxon under discussion and therefore *Nigroboletus roseonigrescens* is proposed as a novel genus and species.

## Materials and Methods

### Collection sites and sampling

Specimens examined were collected at different localities in Guangzhou, Guangdong Province, China, and are deposited in GDGM, ZT (acronyms from Thiers [31]) and ‘‘MG”, which refers to the personal herbarium of Matteo Gelardi. Herbarium numbers are cited for all collections from which morphological features were examined. Author citations follow the Index Fungorum, Authors of Fungal Names (www.indexfungorum.org/authorsoffungalnames.htm). No specific permits were required for the described field studies. The field studies did not involve endangered and protected species.

### Morphological studies

Macroscopic descriptions, macro-chemical reactions (20% KOH, FeSO_4_), habitat notations and associated plant communities were based upon detailed field notes from fresh basidiomes. Color terms in capital letters (e.g. Raw Umber, Plate III) are those of Ridgway [32]. Micro-morphologic features were observed from dried material; sections either were rehydrated in water, 5% KOH or in ammoniacal Congo red. Observation of structures and measurements of anatomical features were performed by mounting preparations in ammoniacal Congo red. Colors and amount of pigmentation were described after examination in water and 5% KOH. Measurements were made at 1000× with a calibrated ocular micrometer (Nikon Eclipse E200 optical light microscope). Spores were measured from the hymenophore of mature basidiomes, dimensions are given as (minimum) average ± standard deviation (maximum), Q = length/width ratio with minimum and maximum values in parentheses, Qm = average quotient (length/width ratio) ± standard deviation, while average spore volume was approximately estimated as a rotation ellipsoid (V = 4/3*(length/2)*((width/2)*width) *π/2 ± standard deviation). The notation [n/m/p] indicates that measurements were made on “n” randomly selected spores from “m” basidiomes of “p” collections. The width of each basidium was measured at the widest part, and the length was measured from the apex (sterigmata excluded) to the basal septum. Metachromatic, cyanophilic and iodine reactions were tested by staining the spores in Brilliant Cresyl blue, Cotton blue and Melzer’s reagent, respectively. Line-drawings of microstructures were made free hand from rehydrated material and based on photomicrographs.

### DNA extraction, PCR amplification and DNA sequencing

Total DNA was extracted from three dry specimens (Table 1) blending a portion of them (about 20 mg) with the aid of a micropestle in 600 μL CTAB buffer (CTAB 2%, NaCl 1.4 M, EDTA pH 8.0 20 mM, Tris-HCl pH 8.0 100 mM). The resulting mixture was incubated for 15 min at 65°C. A similar volume of chloroform: isoamyl alcohol (24:1) was added and carefully mixed with the samples until their emulsion. It was then centrifuged for 10 min at 13.000 g, and the DNA in the supernatant was precipitated with a volume of isopropanol. After a new centrifugation of 15 min at the same speed, the pellet was washed in cold ethanol 70%, centrifuged again for 2 min and dried. It was finally resuspended in 200 μL ddH_2_O. PCR amplification was performed with the primers ITS1F and ITS4 [33, 34] for the nrITS region, while LR0R and LR5 [35] were used to amplify the nrLSU region, reverse of bRPB2-6R2 [36] and bRPB2-7.1R2 (5’ – CCCATNGCYTGYTTVCCCATDGC – 3’) or RPB2-B-F1 and RPB2-B-R [26] for the RNA polymerase II second largest subunit, *rpb2*, RPB1-Af and RPB1-Cr [37] or RPB1-B-F and RPB1-B-R [26] for the RNA polymerase II largest subunit, *rpb1*, and finally EF1-983F and EF1-1567R [38] or EF1-B-F1 and EF-B-R [26] for the translation elongation factor 1-α (*tef1-α*) gene. PCR reactions were performed under a program consisting of a hot start at 95°C for 5 min, followed by 35 cycles at 94°C, 54°C and 72°C (45, 30 and 45 s respectively) and a final 72°C step for 10 min. PCR products were checked in 1% agarose gel, and positive reactions were sequenced with primer ITS4. Chromatograms were checked searching for putative reading errors, and these were corrected. The sequences were deposited in GenBank (Table 1).

**Table 1:** Collections newly sequenced in this study.

Species	GenBank acc. numbers					Source and country
	nrITS	nrLSU	*rpb1*	*rpb2*	*tef1-α*	
*Nigroboletus roseonigrescens*	KT220584	KT220588	KT220591	—	KT220595	GDGM43238 (holotype), CHINA
*Nigroboletus roseonigrescens*	KT220585	KT220589	KT220592	KT220594	KT220596	ZT 13553, CHINA
*Nigroboletus roseonigrescens*	KT220586	KT220590	KT220593	—	—	MG524a, CHINA

### Sequence alignment, data set assembly and phylogenetic analyses

**Fig 1:**
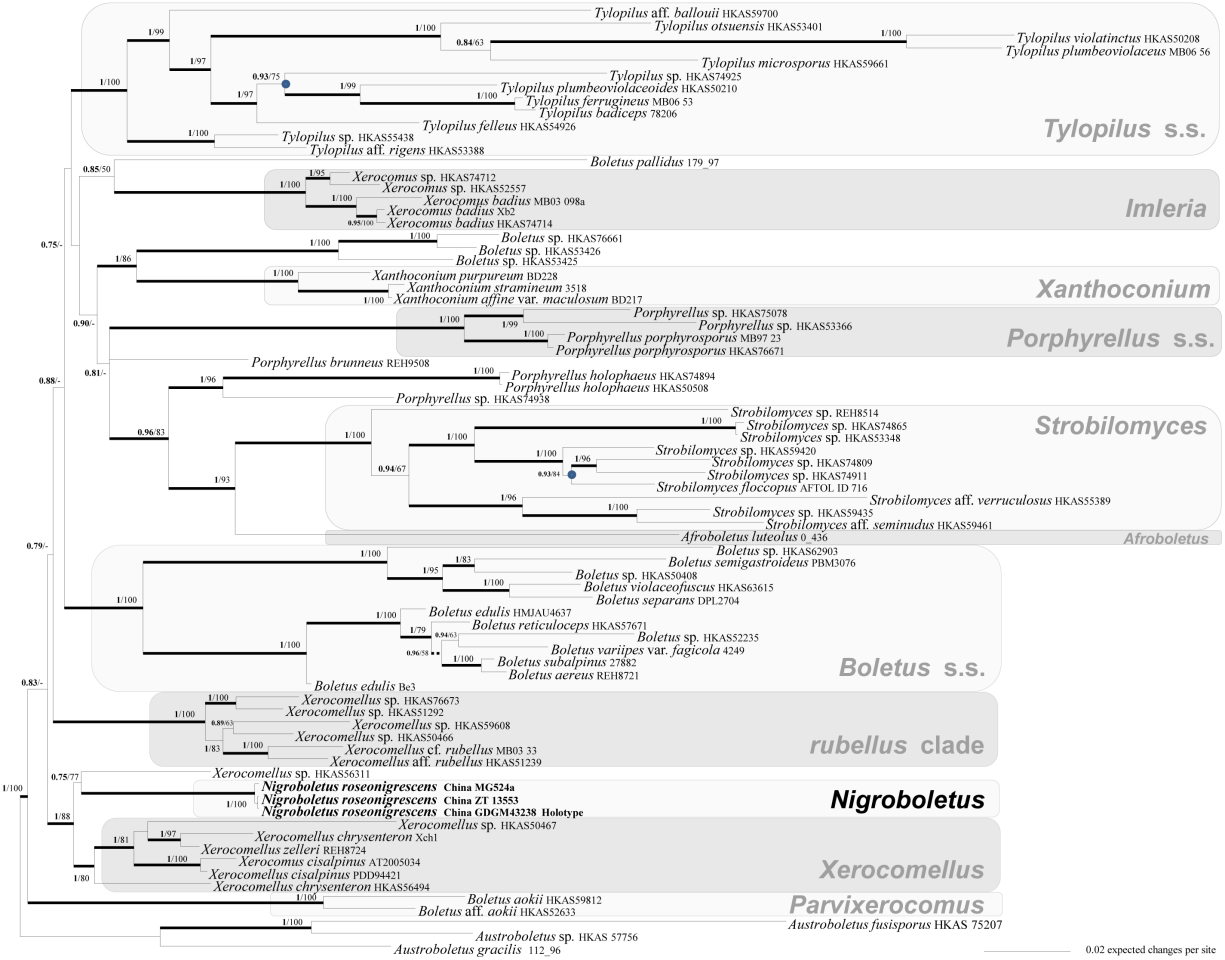
Phylogeny of the Boletoideae based on a Bayesian and Maximum Likelihood Inference analysis of a supermatrix of four nuclear gene regions (nrLSU, *rpb1*, *rpb2* and *tef1-α*). Bayesian posterior probability (BPP) values (in bold) ≥ 0.7 and Maximum Likelihood bootstrap (MLB) values ≥ 50% are shown on the branches. Thickened branches indicate BPP ≥ 0.95 and MLB support ≥ 70%. Dashed branches indicate BPP ≥ 0.95 and MLB bootstrap support < 70%. Nodes that receive BPP < 0.95 but with ≥ 70% MLB are indicated by small black–filled circles. Accession numbers of the sequences retrieved from GenBank refer to Wu et al. (2014, 2015). Newly sequenced collections are in bold.

**Fig 2:**
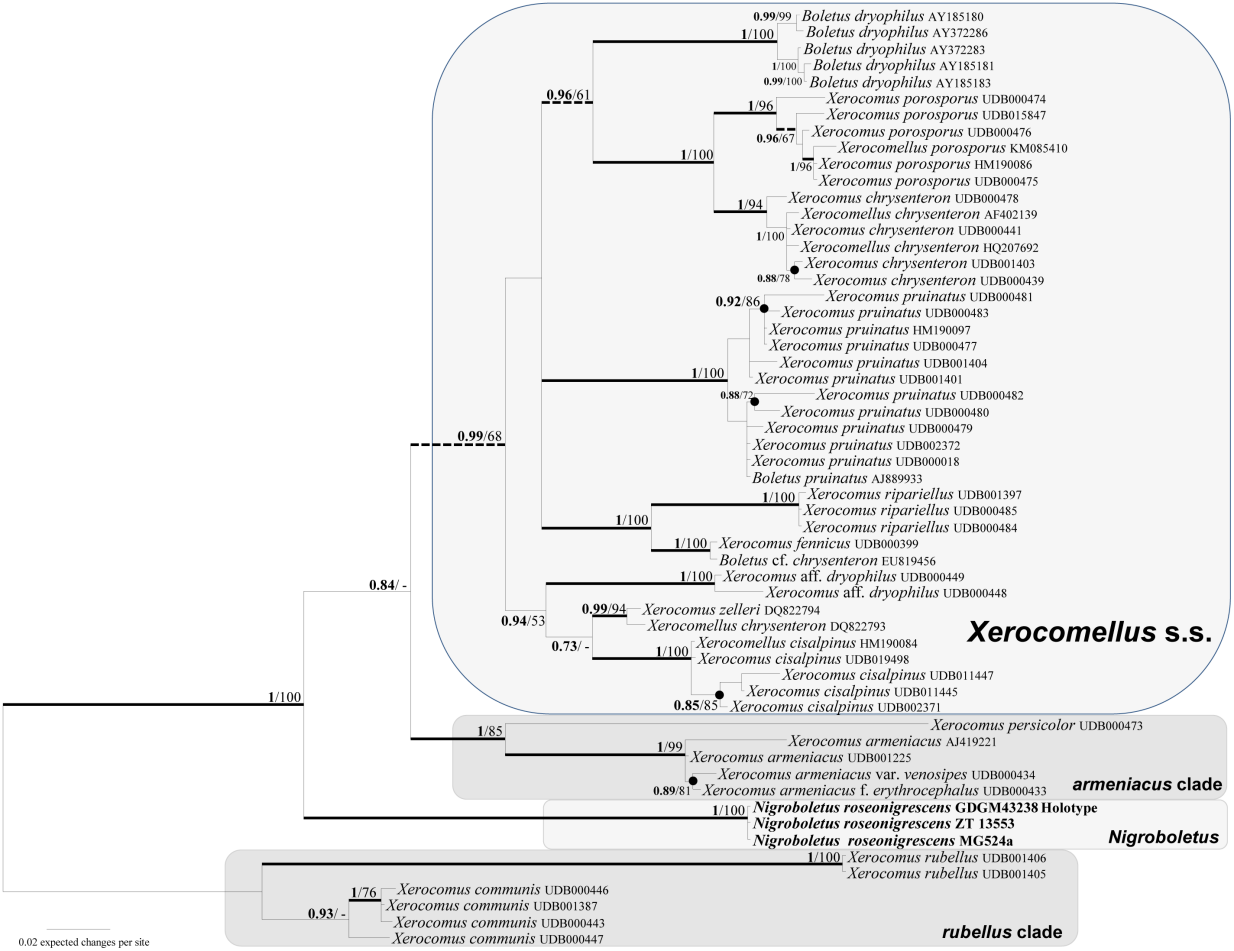
ITS phylogeny restricted to the major clade including *Xerocomellus* and *Nigroboletus*. BPP values (in bold) ≥ 0.7 and MLB values ≥ 50% are shown on the branches. Thickened branches indicate BPP ≥ 0.95 and MLB support ≥ 70%. Dashed branches indicate BPP ≥ 0.95 and MLB bootstrap support < 70%. Nodes that receive BPP < 0.95 but with ≥ 70% MLB are indicated by small black–filled circles. Newly sequenced collections are in bold.

The sequences obtained in this study were checked and assembled using Geneious v5.3 [39] and compared to those available in GenBank database by using the Blastn algorithm. A general combined Maximum Likelihood tree including all the Boletaceae sequences present in GenBank and UNITE (http://unite.ut.ee/) databases was generated to detect the phylogenetic position of our collections in the major clades of Boletaceae as circumscribed by Wu et al. [26] (tree not shown). Consequently, phylogenetic analyses were restricted to the major clade including *Nigroboletus* sequences (subfamily Boletoideae, Figs 1 and 2). Alignments were generated for each ITS, LSU, *rpb1*, *rpb2* and *tef1-α* dataset with MAFFT [40] with default conditions for gap openings and gap extension penalties. Alignments were imported into MEGA 6.06 [41] for manual adjustment. The best-fit substitution model for each alignment was estimated by the Bayesian information criterion (BIC) with jModelTest 2.0 [42] to provide a substitution model for the alignment. GTR+G model was chosen for the ITS alignment, while TrN+G was selected for LSU, 010230+G for *rpb1*, 010023+G for *rpb2* and TIM3ef+G for *tef1-α* alignments. Two phylogenetic analyses were performed: the first large phylogenetic analysis, based on a combined LSU/*rpb1*/*rpb2*/*tef1-α* dataset, was focused on the intergeneric position of *Nigroboletus* in the Boletoideae as delimited by Wu et al. [26]. According to the results by Wu et al. [26], species of *Austroboletus* (Austroboletoideae) were chosen as outgroup taxa for the combined dataset. The second phylogenetic analysis based only on a ITS dataset was restricted to the taxa closely related to *Nigroboletus*: members of the *rubellus* clade (*Xerocomus rubellus* and *X*. *communis*) were selected as outgroup taxa.

Phylogenetic hypotheses were constructed with Bayesian inference (BI) and Maximum likelihood (ML) criteria. The BI was performed with MrBayes 3.2.2 [43] with four incrementally heated simultaneous Monte Carlo Markov chains (MCMC) run for 10 000 000 generations, under the selected evolutionary model. Trees were sampled every 1000 generations, resulting in overall sampling of 10 001 trees; the first 2500 trees (25%) were discarded as burn-in. For the remaining trees, a majority rule consensus tree showing all compatible partitions was computed to obtain estimates for Bayesian posterior probabilities (BPP). ML estimation was performed with RAxML 7.3.2 [44] with 1000 bootstrap replicates [45] using the GTRGAMMA algorithm to perform a tree inference and search for optimal topology. Support values from bootstrapping runs (MLB) were mapped on the globally best tree using the ‘‘-fa”option of RAxML and ‘‘-b 12345” as a random seed to invoke the novel rapid bootstrapping algorithm. BI and ML analyses were run on the CIPRES Science Gateway web server [46]. Only BPP values exceeding 0.75 and MLB over 50% are reported in the resulting trees (Figs 1 and 2). Branch lengths were estimated as mean values over the sampled trees. Alignments and phylogenetic trees are available at TreeBASE (www.treebase.org, submission number S17886).

### Nomenclature

The electronic version of this article in Portable Document Format (PDF) in a work with an ISSN or ISBN will represent a published work according to the International Code of Nomenclature for algae, fungi, and plants, and hence the new names contained in the electronic publication of a PLOS ONE article are effectively published under that Code from the electronic edition alone, so there is no longer any need to provide printed copies.

In addition, new names contained in this work have been submitted to MycoBank, from where they will be made available to the Global Names Index. The unique MycoBank number can be resolved and the associated information viewed through any standard web browser by appending the MycoBank number contained in this publication to the prefix www.mycobank.org/MB. The online version of this work is archived and available from the following digital repositories: PubMed Central and LOCKSS.

## Results

### Molecular analysis

Both Bayesian and Maximum likelihood analyses produced the same topology; therefore only the Bayesian trees with both BPP and MLB values are shown (Figs 1 and 2). The combined data matrix (focused on the Boletoideae) comprised 256 sequences (including 247 from GenBank). corresponding to 76 collections. The three newly sequenced collections of *Nigroboletus roseonigrescens*, together with *Xerocomellus* sp. HKAS56311 (BPP = 0.75; MLB = 77), occupy a sister position (BPP = 1; MLB = 88) to the clade consisting of *Xerocomellus* species (BPP = 1; MLB = 80) (Fig 1). The ITS data matrix comprised 57 sequences (including 16 from GenBank and 38 from UNITE). In the ITS analysis *Nigroboletus* is clearly distinct from *Xerocomellus* and from two *Xerocomellus* species, *X*. *armeniacus* and *X*. *persicolor*, which form an independent clade (here referred as the *armeniacus* clade) (Fig 2).

### Taxonomy

#### Nigroboletus Gelardi, Vizzini, E. Horak, T.H. Li & Ming Zhang, gen. nov. [urn:lsid:mycobank.org:names: MB 813026]

Etymology: the generic epithet “nigro” (black) is derived from Latin adjective *niger* and refers to the conspicuous, overall blackening of the tissues when injured.

Original diagnosis: Basidiome stipitate–pileate with tubular hymenophore, epigeal, evelate, medium–small sized; pileus convex to applanate, subtomentose to glabrous; hymenophore very thin, poroid, adnate to subdecurrent, yellow to olive–yellow; stipe solid, dry, smooth to minutely pruinose-punctate, reticulation absent; context firm, yellowish; tissues turning dull grayish to blackish throughout when injured or exposed; taste mild; spore print olive–brown; spores smooth, broadly ellipsoid to subovoid; pleuro–, cheilo—and caulocystidia present; pileipellis consisting of subparallel to loosely interwoven erect hyphae; hymenophoral trama bilateral–divergent of the *Boletus*–type or intermediate between the *Boletus*–type and the *Phylloporus*–type; lateral stipe stratum of the boletoid type; clamp connections absent; ontogenetic development gymnocarpic.

Typus generis: *Nigroboletus roseonigrescens* Gelardi, Vizzini, E. Horak, T.H. Li & Ming Zhang.

#### Nigroboletus roseonigrescens Gelardi, Vizzini, E. Horak, T.H. Li & Ming Zhang, sp. nov. (Figs 3–5) [urn:lsid:mycobank.org:names: MB 813027]

Etymology: the specific epithets “roseo” (pink) and “nigrescens” (blackening) are derived from Latin and refer to the pinkish color of pileus and stipe which turn blackish when damaged.

**Fig 3:**
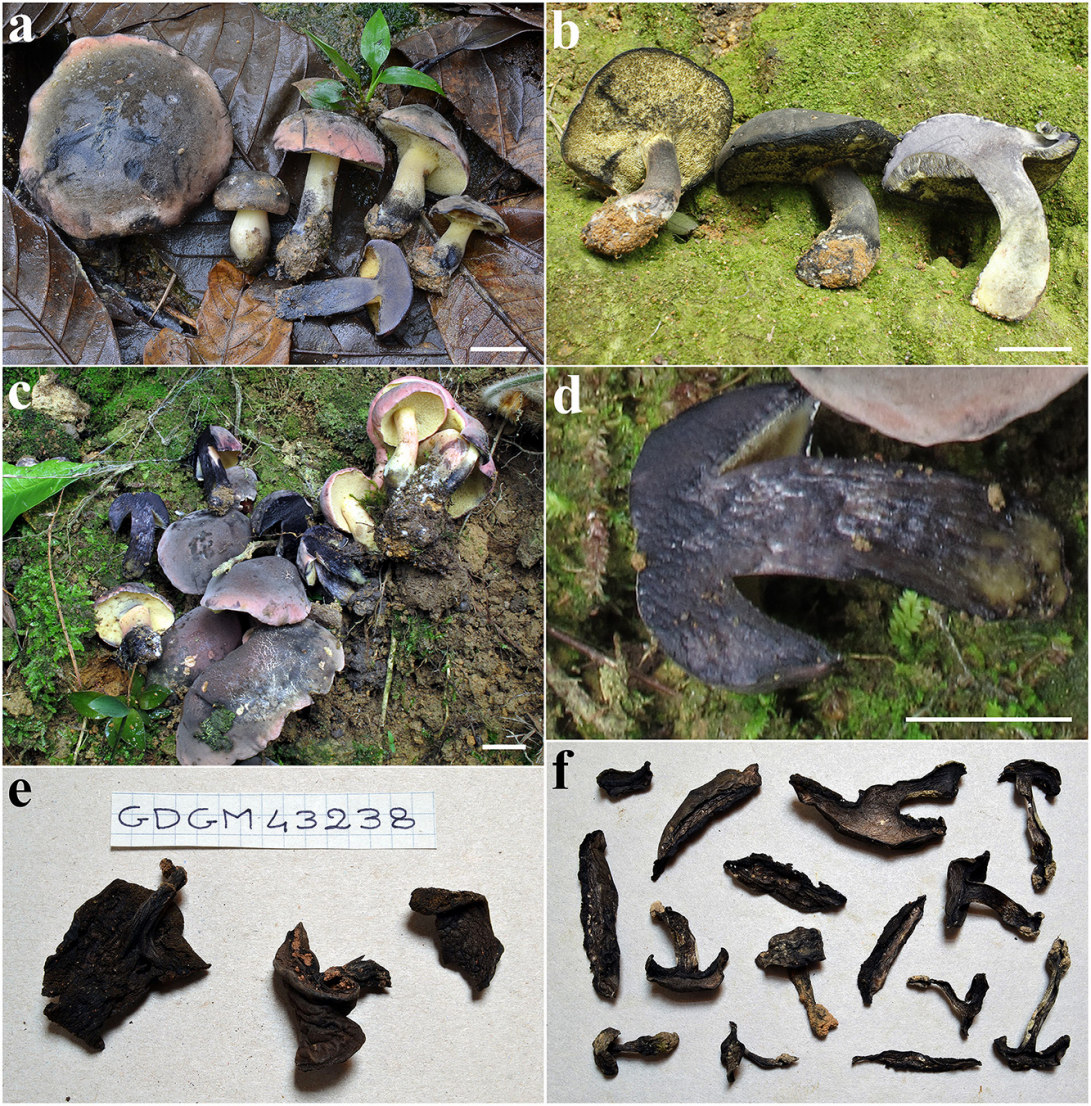
*Nigroboletus roseonigrescens*. **a**–**d** Fresh basidiomes. **a**. MG524a. **b**. GDGM43238 (holotype). **c**. GDGM42430. **d**. MG524a, blackening context. **e**–**f** Dried collections. **e**. GDGM43238 (holotype). **f**. MG524a. *Scale bars* = 2 cm.

**Fig 4:**
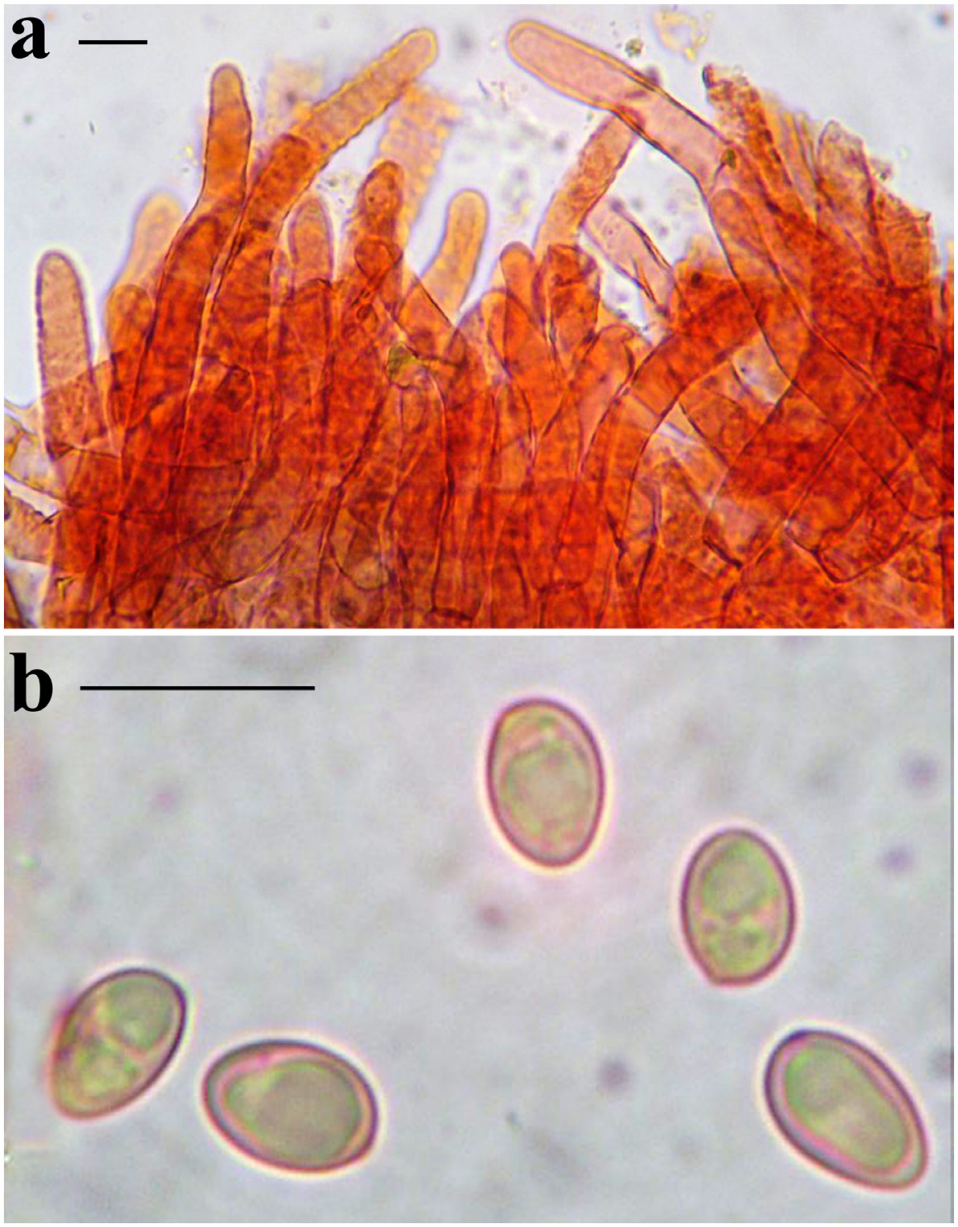
*Nigroboletus roseonigrescens*. Microscopic features (from MG524a). **a**. Pileipellis in ammoniacal Congo red. **b**. Spores in ammoniacal Congo red. *Scale bars* = 10 μm. Photos by M. Gelardi.

**Fig 5:**
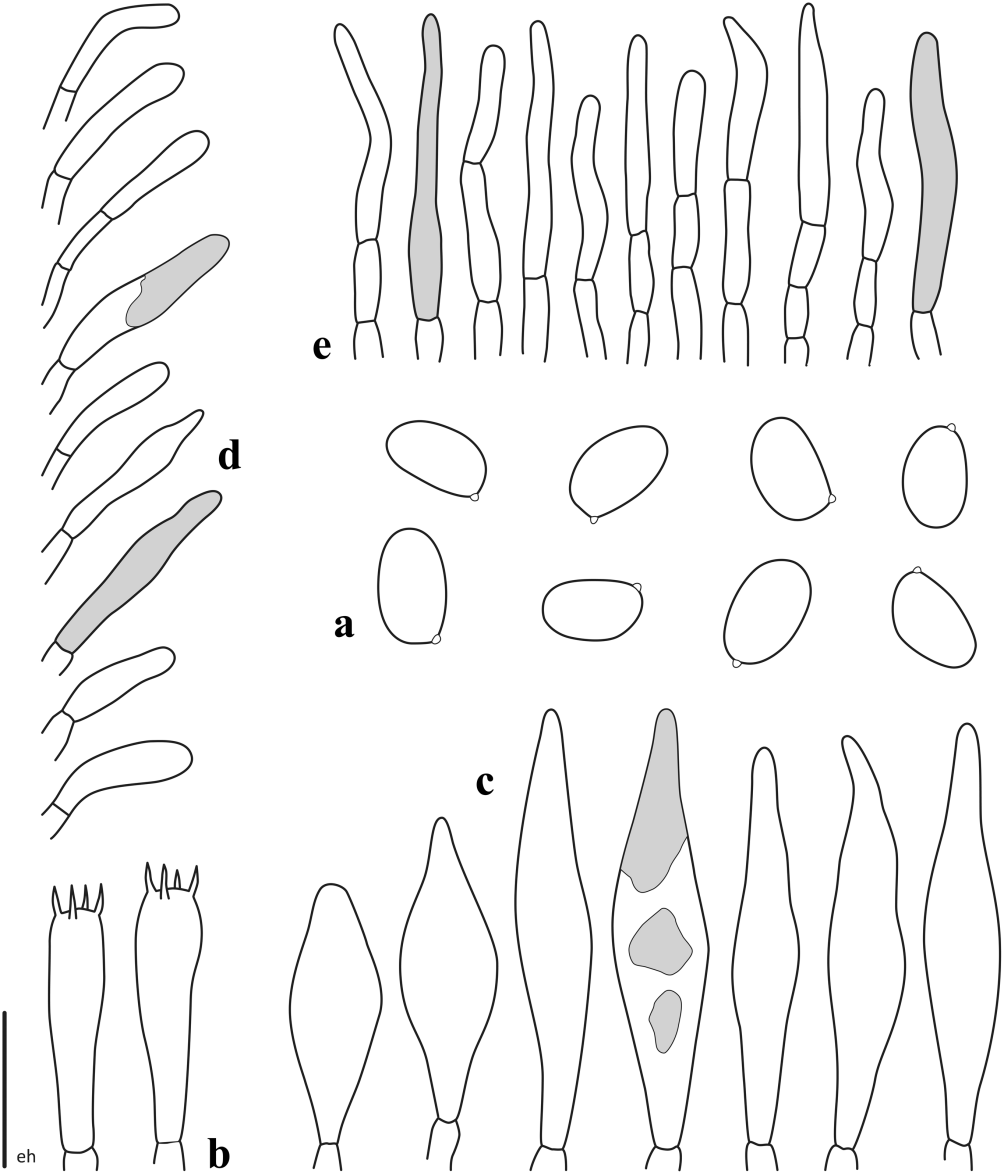
*Nigroboletus roseonigrescens*. Microscopic features (from ZT 13553). **a**. Spores. **b**. Basidia. **c**. Cheilo- and pleurocystidia. **d**. Caulocystidia. **e**. Elements of pileipellis. *Scale bar*: **a** = 10 μm; **b**, **c** = 20 μm; **d**, **e** = 40 μm. Drawings by E. Horak.

Original diagnosis: Basidiomes characterized by xerocomoid habit, pinkish pileus, yellowish to pinkish-orange stipe surface, white basal mycelium, yellowish context, tissues turning dull grayish to blackish throughout when injured or exposed, broadly ellipsoid to subovoid, smooth spores, pileipellis consisting of subparallel to loosely interwoven erect hyphae with subtle granular or zebra–pattern epiparietal encrustations and presence of congophilous plaques on cystidial wall.

Holotype: CHINA, Guangdond Province, Guangzhou, Mount Huolu, Tianlu Lake Forest Park, 23°13'35''N 113°110'05''E, 360 m, 14 Sep 2012, M. Zhang (GDGM 43238; isotype GDGM 43237 and MG609a).

Detailed description: Basidiomes small to medium sized. Pileus (3.0–) 3.2–8.0 (–10.0) cm broad, at first hemispherical then persistently convex and finally broadly pulvinate-flattened, moderately fleshy, firm at the beginning but progressively softer with age; margin steady to faintly wavy-lobed, initially involute then curved downwards and eventually plane or even uplifted, not or only a little extending beyond the tubes; surface matt, dry, very finely tomentose in the early stage of development but later smooth and glabrous, not cracked; cuticle evenly pink or pastel rose (Flesh Pink, Chatenay Pink, Venetian Pink, Coral Pink, Plate XIII), slowly staining grayish (Pale Mouse Gray, Pale Quaker Drab, Plate LI) to dull sooty brown (Raw Umber, Plate III; Blackish Brown, Plate XLV) and finally blackening (Sooty Black, Plate LI) on handling, when injured or even at the slightest contact with rain drops (Fig 3a–3c); subcuticular layer pale cream. Tubes at first thin then broader and always shorter than the thickness of the pileus context, particularly in young specimens (up to 1 cm long), adnate to subdecurrent on stipe, straw yellow (Martius Yellow, Plate IV) to bright yellow (Lemon Yellow, Plate IV) and finally olive yellow (Javel Green, Plate V), bruising dark gray (Deep Mouse Gray, Quaker Drab, Plate LI) to blackish (Sooty Black, Plate LI) when cut. Pores initially concave or even, later with a convex–anticlinal surface, small at first then gradually wider (up to 1.5 mm diam), simple, roundish to angular and radially arranged, concolorous with tubes, turning dark grayish (Deep Mouse Gray, Quaker Drab, Plate LI) and quickly becoming blackish (Sooty Black, Plate LI) when bruised (Fig 3b). Stipe (2.0–) 3.2–6.0 (–7.0) × (0.8–) 1.0–1.7 (–2.0) cm, shorter than or more often as long as the pileus diameter at maturity, central to slightly off–center, solid, firm, dry, straight or curved, cylindrical or fusiform to more frequently swollen towards the base, sometimes squashed, ending with a roundish base, not rooting; surface devoid of reticulum, smooth at apex but finely pruinose–punctate elsewhere, evelate; at first uniformly straw yellow (Martius Yellow, Plate IV) to bright yellow (Lemon Yellow, Plate IV), later pinkish (Flesh Pink, Chatenay Pink, Venetian Pink, Plate XIII) to pale orange-yellow (Orange, Plate III), quickly turning gray-blackish (Blackish Mouse Gray, Sooty Black, Plate LI) when pressed; basal mycelium white (White, Plate LIII), rhizomorphs brownish (Sayal Brown, Plate XXIX). Context firm when young, later soft in the pileus, up to 1.7 cm thick in the central zone, a little more fibrous in the stipe, yellowish (Martius Yellow, Plate IV) to pale cream (Maize Yellow, Plate IV) in the pileus, of a deeper yellow in the stipe but dirty yellowish (Apricot Yellow, Plate IV) and occasionally with orange hues (Ochraceous–Orange, Plate XV) towards the base; evenly but slowly turning pale pinkish (Light Pinkish Cinnamon, Plate XXIX) on exposure especially in the pileus, then grayish–violaceous (Dark Purple–Drab, Table XLV) and finally becoming blackish (Sooty Black, Plate LI) (Fig 3d); subhymenophoral layer pale cream (Maize Yellow, Plate IV); dried material sordid blackish (Sooty Black, Plate LI) throughout (Fig 3e and 3f). Odor faintly fruity, agreeable. Taste mild.

Macrochemical reactions: 20% KOH: blackish on pileus, hymenophore and stipe surface, dull ochraceous on context; FeSO_4_: no reaction.

Spores [157/7/4] (5.2–) 7.4 ± 0.77 (–10.1) × (4.0–) 4.7 ± 0.33 (–6.4) μm, Q = (1.27–) 1.30–2.06 (–2.15), Qm = 1.57 ± 0.15, V = 86 ± 20 μm³, asymmetric, broadly ellipsoid to subovoid, less frequently ellipsoid in side view, broadly ellipsoid to ovoid or even subglobose in face view, smooth, with a short apiculus and without suprahilar depression, adaxially applanate, apex rounded, thin-walled (0.3–0.5 μm), straw yellow coloured in water and 5% KOH, having one large or occasionally two oil droplets when mature, inamyloid to very slighty dextrinoid, acyanophilic and showing an orthochromatic reaction (Figs 4b and 5a). Basidia 26–44 (–48) × 6–10 (–12) μm (n = 32), cylindrical to cylindrical–clavate, rarely truly clavate, moderately thick–walled (0.5–1.0 μm), predominantly 4–spored but frequently also 1–, 2– or 3–spored, with relatively long sterigmata (2–6 μm), hyaline to very pale yellowish in water and hyaline to yellowish in KOH, usually containing straw-yellow oil guttules, without basal clamps, inamyloid (Fig 5b); basidioles cylindrical to clavate, about the same size as basidia. Cheilocystidia 30–59 (–63) × 7–16 (–18) μm (n = 47), frequent, moderately slender, projecting straight to sometimes flexuous, fusiform to ventricose–fusiform or sublageniform, occasionally clavate or mucronate, with short neck and rounded or rarely pointed tip, smooth, moderately thick-walled (0.6–1.2 μm), with a brownish to dark brown plasmatic pigment in water and KOH, occasionally hyaline, reddish-brown (dextrinoid) in Melzer’s, without epiparietal encrustations but sometimes with brownish to dark brown, congophilous plaques (Fig 5c). Pleurocystidia 31–66 (–68) × 10–18 μm (n = 29), color, size, shape and chemical reactions as in cheilocystidia, very frequent (Fig 5c). Pseudocystidia present but rare, rising from the oleiferous hyphae of the inner hymenium, up to 125 × 12 μm (coll. MG524a). Pileipellis (Figs 4a and 5e) a trichoderm consisting of erect subparallel to loosely interwoven (palisadoderm), filamentous and sinuous, unbranched hyphae usually not constricted at septa, tending to remain erect or to be repent only in the outermost layer, not embedded in gelatinous matter; terminal elements (22–) 31–150 (–156) × 6–15 (–18) μm (n = 36), slender, cylindrical, only rarely short, acorn–shaped or bullet–shaped, occasionally cystidioid, apex rounded–obtuse and enlarged or tapered, moderately thick–walled (0.5–1.0 μm), with a brownish–yellow to dark brown plasmatic pigment in water and KOH and reddish-orange (inamyloid to weakly dextrinoid) in Melzer’s, smooth to more often ornamented with subtle granular or zebra–pattern epiparietal encrustations; subterminal elements similar in shape, color and dimensions with terminal ones. Stipitipellis a texture of slender, subparallel to loosely intermingled and longitudinally running, smooth walled, adpressed hyphae, 2–7 (11) μm wide, some hyaline to pale yellow but others with brownish to dark brown plasmatic pigment in water and KOH; the stipe apex distinctly covered with caulobasidioles, spore-bearing caulobasidia (mainly 4–, 2– and 3–spored but also 1–spored, 29–37 × 8–9 μm (n = 4), sterigmata 3–6 μm long) and very frequent projecting caulocystidia (Fig 5d) similar in shape, size, color and chemical reactions to the hymenial ones, sporadically with secondary septa, (32) 38–72 (86) × (6) 8–15 (18) μm (n = 16), moderately thick–walled (up to 1.2 μm); elsewhere the stipe surface exhibits a layer of sterile cystidioid elements. Lateral stipe stratum under the caulohymenium present and well differentiated from the stipe trama, of the “boletoid type”, at the stipe apex a 40–100 (160) μm thick layer consisting of divergent, inclined and running towards the external surface, loosely intermingled and sparingly branched hyphae remaining separate from each other and faintly to distinctly embedded in a gelatinous substance; the stratum reducing during development and finally disappearing at maturity. Stipe trama composed of densely arranged, loosely to strongly interwoven, filamentous, smooth hyphae, 4–22 μm broad, some hyaline and inamyloid, others (oleiferous hyphae) with a brownish to dark brown plasmatic pigment in water and KOH and dextrinoid in Melzer’s. Hymenophoral trama bilateral divergent of the “*Boletus*–type” or less frequently intermediate between the “*Boletus*–type” and the “*Phylloporus*–type”, with moderately to distinctly divergent and loosely arranged, gelatinized hyphae, lateral strata hyphae often branched, constricted at septa, in transversal section remaining separate and (1–) 2–8 (–10) μm apart, some hyaline to very pale yellowish in water and KOH and inamyloid in Melzer’s, others (oleiferous hyphae) with a brownish to dark brown plasmatic pigment in water and KOH and dextrinoid in Melzer’s; lateral strata (15–) 20–50 (–60) μm thick, mediostratum (10–) 40–50 μm thick, consisting of a tightly adpressed, not gelatinized bundle of hyphae, 2–12 μm wide; in Congo red the mediostratum is darker than the lateral strata. Clamp connections absent (but a solitary clamp has been observed in the peripheral layer of the stipe trama). Hyphal system monomitic.

Habitat: Gregarious or scattered, growing in clayey soil among litter in tropical montane and lowland mixed forests dominated by *Castanopsis fissa* (Champion ex Bentham) Rehder & E. H. Wilson, *Castanea henryi* (Skan) Rehder & E. H. Wilson and *Lithocarpus* sp. with the presence of *Schima superba* Gardner & Champ. and *Litsea rotundifolia* var. *oblongifolia* (Nees) C.K. Allen, late spring to early autumn.

Known distribution: So far known only from south–eastern China (Guangdong Province), distribution limits unknown.

Examined material: CHINA, GUANGDONG PROVINCE, Guangzhou, Mount Huolu, Tianlu Lake Forest Park, 23°13'35''N, 113°110'05''E, 360 m, with *Castanopsis fissa* and *Schima superba*, 14 Sep 2012, M. Zhang (GDGM 43238, **holotype**; GDGM 43237 and MG609a, **isotypes**); same location, with *Castanopsis* sp., 11 Aug 2014, E. Horak (ZT 13553, dupl. in herb. MG674a); Guangzhou, Mount Baiyun, 23°10'00'' N, 113°17'00''E, 280 m, with *Castanea henryi*, *Castanopsis fissa*, *Lithocarpus* sp. and *Litsea rotundifolia* var. *oblongifolia*, 02 Jul 2013, P. Li and M. Gelardi, (MG524a); same location but different microhabitat, 12 May 2012, W.–Q. Deng and P. Li (GDGM42430, dupl. in herb. MG677a).

## Discussion

### *Nigroboletus* phylogeny and intergeneric relationships

The combined and ITS molecular analyses (Figs 1 and 2) clearly indicate that *Nigroboletus* represents a new and independent phyletic line within the subfamily Boletoideae in sister position to the genus *Xerocomellus* Šutara, typified by *X*. *chrysenteron* (Bull.) Šutara. *Nigroboletus* shares with *Xerocomellus* the medium–small size, dry subtomentose to velutinous pileus surface, wide, roundish to angular and radially arranged yellowish to olive–yellow pores, stipe without reticulate ornamentation and microscopically the trichodermic pileipellis with encrusting pigment, fertile stipe surface and the similar hymenophoral trama structure. *Xerocomellus* is morphologically delimited from *Nigroboletus* by the longer, ellipsoid–fusoid to subfusoid spores with more or less pronounced suprahilar depression, the lateral stipe stratum usually absent or, whenever present, very thin (up to 40 μm thick at most), the absence of congophilous plaques and the occurrence in North boreal and temperate habitats [47]. Apart from the comparative features listed above with respect to *Xerocomellus* s.l., the *armeniacus* clade further shares with *Nigroboletus* the presence of congophilous plaques on hyphal surface, whereas the differential characters also include the bright yellow–ochraceous to orange–yellow and unchangeable context in the stipe base and the dark blue–green reaction with FeSO_4_ on pileus surface and in the stipe base context [47, 48].

### Taxonomic circumscription of *N*. *roseonigrescens*

Given the geographical range of the tropical belt and despite recent advances in mycological research, our present knowledge of tropical boletes is still scarce. As a result, even though boletes are thriving in pantropical habitats, only a few of them have been recognized and named to date.

The new taxon *Nigroboletus roseonigrescens*, originating from tropical south–eastern China, revealed to be morphologically and phylogenetically unique amongst Boletaceae and exhibits several clear–cut features. The diagnostic macromorphological features of *N*. *roseonigrescens* include the following combination: (1) Small to medium–small sized basidiomes, (2) pastel pink pileus, (3) very thin tubes, (4) yellow to pinkish–orange pruinose stipe, (5) pale cream to yellowish context, (6) white basal mycelium. Noteworthy is the color change of the tissues to blackish, which is likely to be addressed to the presence of abundant oleiferous hyphae and cystidia with dark brown, dextrinoid content spreading out after bruising or sectioning. Other distinctive features are: the shape and size of the spores as the small, broadly ellipsoid to subovoid outline without suprahilar depression is unlike most of Boletaceae and is conversely more reminiscent of the spore morphology found in Paxillaceae [49]; the presence of congophilous plaques on cystidial wall. *Xerocomellus* sp. (HKAS56311) from China might represent an additional species of *Nigroboletus* (Fig 1), but more material is required to elucidate the status of the taxon.

After checking all relevant accounts to the knowledge of boletes of Guangdong and neighboring areas [25, 50–61] and monographic works focused on boletoid fungi worldwide [48, 62–72], it appears evident there is no known taxon that can be referred to *N*. *roseonigrescens*.

The new species seems to be fairly common and abundant but at present it is premature to determine the frequency and bio–geographical boundaries of *Nigroboletus*, even though a south–eastern Asian distribution in tropical and subtropical regions appears quite likely. We therefore expect *N*. *roseonigrescens* to be found at other localities in southern China and adjacent regions.

## References

[1] Li YC, Yang ZL, Tolgor B (2009) Phylogenetic and biogeographic relationships of *Chroogomphus* species as inferred from molecular and morphological data. Fungal Divers 38:85–104.

[2] Li YC, Feng B, Yang ZL (2011) *Zangia*, a new genus of Boletaceae supported by molecular and morphological evidence. Fungal Divers 49(1): 125–143, 10.1007/s13225-011-0096-y

[3] Li YC, Li F, Zeng NK, Cui YY, Yang ZL (2014a) A new genus *Pseudoaustroboletus* (Boletaceae, Boletales) from Asia as inferred from molecular and morphological data. Mycol Prog 13(4): 1207–1216, 10.1007/s11557-014-1011-1

[4] Li YC, Ortiz–Santana B, Zeng NK, Feng B, Yang ZL (2014b) Molecular phylogeny and taxonomy of the genus *Veloporphyrellus* . Mycologia 106(2): 291–306, 10.3852/106.2.291 24782497

[5] Gelardi M, Vizzini A, Ercole E, Voyron S, Wu G, Liu XZ (2012) *Strobilomyces echinocephalus* sp. nov. (Boletales) from south–western China, and a key to the genus *Strobilomyces* worldwide. Mycol Prog 12(3): 575–588, 10.1007/s11557-012-0865-3

[6] Gelardi M, Vizzini A, Ercole E, Voyron S, Sun JZ, Liu XZ (2013) *Boletus sinopulverulentus*, a new species from Shaanxi Province (central China) and notes on *Boletus* and *Xerocomus* . Sydowia 65(1): 45–57, 10.12905/0380.sydowia65(1)2013-0045

[7] Gelardi M, Vizzini A, Horak E, Ercole E, Voyron S, Wu G, 2014: *Paxillus orientalis* sp. nov. (Paxillaceae, Boletales) from south-western China based on morphological and molecular data and proposal of the new subgenus *Alnopaxillus* . Mycol Prog 13(2): 333–342, 10.1007/s11557-013-0919-1

[8] Li HB, Wei HL, Peng HZ, Ding HM, Wang LL, He L, et al (2013) *Boletus roseoflavus*, a new species of *Boletus* in section *Appendiculati* from China. Mycol Prog 13(1): 21–31, 10.1007/s11557-013-0888-4

[9] Orihara T, Smith ME, Ge ZW, Maekawa N (2012) *Rossbeevera yunnanensis* (Boletaceae, Boletales), a new sequestrate species from southern China Mycotaxon 120: 139–147, 10.5248/120.139

[10] Wang SR, Wang Q, Wang DL, Li Y (2014) *Gastroboletus thibetanus*: a new species from China. Mycotaxon 129: 79–83, 10.5248/129.79

[11] Yan WJ, Li TH, Zhang M, Li T (2013) *Xerocomus porophyllus* sp. nov., morphologically intermediate between *Phylloporus* and *Xerocomus* . Mycotaxon 124: 255–262, 10.5248/124.255

[12] Zeng NK, Cai Q, Yang ZL (2012) *Corneroboletus*, a new genus to accommodate the southeastern Asian *Boletus indecorus* . Mycologia 104(6): 1420–1432, 10.3852/11-326 22684293

[13] Zeng NK, Tang LP, Li YC, Tolgor B, Zhu XT, Zhao Q, et al (2013) The genus *Phylloporus* (Boletaceae, Boletales) from China: morphological and multilocus DNA sequence analyses. Fungal Divers 58(1): 73–101, 10.1007/s13225-012-0184-7

[14] Zeng NK, Liang ZQ, Yang ZL (2014a) *Boletus orientialbus*, a new species with white basidioma from subtropical China. Mycoscience 55(3): 159–163, 10.1016/j.myc.2013.07.004

[15] Zeng NK, Wu G, Li YC, Liang ZQ, Yang ZL (2014b) *Crocinoboletus*, a new genus of Boletaceae (Boletales) with unusual boletocrocin polyene pigments. Phytotaxa 175(3): 133–140, 10.11646/phytotaxa.175.3.2

[16] Zeng NK, Su MS, Liang ZQ, Yang ZL (2015) A geographical extension of the North American genus *Bothia* (Boletaceae, Boletales) to East Asia with a new species *B*. *fujianensis* from China. Mycol Prog 14, 10.1007/s11557-014-1015-x

[17] Zhang M, Li TH, Bau T, Song B (2012) A new species of *Xerocomus* from Southern China. Mycotaxon 121: 23–27, 10.5248/121.23

[18] Zhang M, Li TH, Song B (2014) A new slender species of *Aureoboletus* (Boletaceae) from southern China. Mycotaxon 128: 195–202, 10.5248/128.195

[19] Zhang M, Li TH, Xu J, Song B (2015) A new violet brown *Aureoboletus* (Boletaceae) from Guangdong of China. Mycoscience, 10.1016/j.myc.2015.02.002

[20] Zhao K, Wu G, Feng B, Yang ZL (2014a) Molecular phylogeny of *Caloboletus* (Boletaceae) and a new species in East Asia. Mycol Prog 13(4): 1127–1136, 10.1007/s11557-014-1001-3

[21] Zhao K, Wu G, Yang ZL (2014b) A new genus, *Rubroboletus*, to accommodate *Boletus sinicus* and its allies. Phytotaxa 188(2): 61–77, 10.11646/phytotaxa.188.2.1

[22] Zhu XT, Li YC, Wu G, Feng B, Zhao K, Gelardi M, et al (2014) The genus *Imleria* (Boletaceae) in East Asia. Phytotaxa 191(1): 81–98, 10.11646/phytotaxa.191.1.5

[23] Wu G, Zhao K, Li YC, Zeng NK, Feng B, Halling RE, et al (2015) Four new genera of the fungal family Boletaceae. Fungal Divers, 10.1007/s13225-015-0322-0

[24] Feng B, Xu J, Wu G, Zeng NK, Li YC, Tolgor B, et al (2012) DNA Sequence Analyses Reveal Abundant Diversity, Endemism and Evidence for Asian Origin of the Porcini Mushrooms. PLoS ONE 7(5): e37567, 10.1371/journal.pone.0037567 22629418 PMC3356339

[25] Zang M, Li XJ, He YS (2013) Boletaceae (II) Flora Fungorum Sinicorum 44, Science Press, Beijing (in Chinese).

[26] Wu G, Feng B, Xu JP, Zhu XT, Li YC, Zeng NK, et al (2014) Molecular phylogenetic analyses redefine seven major clades and reveal 22 new generic clades in the fungal family Boletaceae. Fungal Divers 69: 93–115, 10.1007/s13225-014-0283-8

[27] Aime MC, Brearley FQ (2012) Tropical fungal diversity: closing the gap between species estimates and species discovery. Biodivers Conserv 21(9): 2177–2180, 10.1007/s10531-012-0338-7

[28] Hawksworth DL (2012) Global species numbers of fungi: Are tropical studies and molecular approaches contributing to a more robust estimate?. Biodivers Conserv 21(9): 2425–2433, 10.1007/s10531-012-0335-x

[29] Tedersoo L, Smith ME (2013) Lineages of ectomycorrhizal fungi revisited: Foraging strategies and novel lineages revealed by sequences from belowground. Fungal Biol Rev 27(3–4): 83–99, 10.1016/j.fbr.2013.09.001

[30] Nuhn ME, Binder M, Taylor AFS, Halling RE, Hibbett DS (2013) Phylogenetic overview of the Boletineae. Fungal Biol 117(7–8): 479–511, 10.1016/j.funbio.2013.04.008 23931115

[31] Thiers B (2015) (continuously updated). Index Herbariorum: a global directory of public herbaria and associated staff. New York botanical garden’s virtual herbarium. http://sweetgum.nybg.org/ih/

[32] Ridgway R (1912) Color standards and color nomenclature. Self-published, Washington D.C.

[33] White TJ, Bruns T, Lee S, Taylor JW (1990) Amplification and direct sequencing of fungal ribosomal RNA genes for phylogenetics In: Innis MA, Gelfand DH, Sninsky JJ, White TJ, eds. PCR protocols: a guide to methods and applications. Academic Press Inc., New York: 315–322.

[34] Gardes M, Bruns TD (1993) ITS primers with enhanced specificity for basidiomycetes—application to the identification of mycorrhizae and rusts. Mol Ecol 2: 113–118, 10.1111/j.1365-294X.1993.tb00005.x 8180733

[35] Vilgalys R, Hester M (1990) Rapid genetic identification and mapping of enzymatically amplified ribosomal DNA from several *Cryptococcus* species. J Bacteriol 172: 4238–4246. 2376561 10.1128/jb.172.8.4238-4246.1990 PMC213247

[36] Matheny PB, Wang Z, Binder M, Curtis JM, Lim YW, Nilsson RH, et al (2007) Contributions of rpb2 and tef1 to the phylogeny of mushrooms and allies (Basidiomycota, Fungi). Mol Phylogen Evol 43: 430–451, 10.1016/j.ympev.2006.08.024 17081773

[37] Matheny PB, Liu YJJ, Ammirati JF, Hall BD (2002) Using RPB1 sequences to improve phylogenetic inference among mushrooms (*Inocybe*, Agaricales). Am J Bot 89: 688–698, 10.3732/ajb.89.4.688 21665669

[38] Rehner SA, Buckley E (2005) A *Beauveria* phylogeny inferred from nuclear ITS and EF1-α sequences: evidence for cryptic diversification and links to *Cordyceps* teleomorphs. Mycologia 97: 84–98, 10.3852/mycologia.97.1.84 16389960

[39] Drummond AJ, Ashton B, Cheung M, Heled J, Kearse M, Moir R, et al. (2010) Geneious 5.3. www.geneious.com

[40] Katoh K, Misawa K, Kuma K, Miyata T (2002) MAFFT: a novel method for rapid multiple sequence alignment based on fast Fourier transform. Nucleic Acids Res 30: 3059–3066, 10.1093/nar/gkf436 12136088 PMC135756

[41] Tamura K, Stecher G, Peterson D, Filipski A, Kumar S (2013) MEGA6: Molecular Evolutionary Genetics Analysis version 6.0. Mol Biol Evol 30: 2725–2729. 10.1093/molbev/mst197 24132122 PMC3840312

[42] Darriba D, Taboada GL, Doallo R, Posada D (2012) jModelTest 2: more models, new heuristics and parallel computing. Nat Methods 9(8): 772. 10.1038/nmeth.2109 PMC4594756 22847109

[43] Ronquist F, Teslenko M, van der Mark P, Ayres DL, Darling A, Höhna S, et al (2012) MrBayes 3.2: efficient Bayesian phylogenetic inference and model choice across a large model space. Syst Biol 61: 539–542. 10.1093/sysbio/sys029 22357727 PMC3329765

[44] Stamatakis A (2006) RAxML-VI-HPC: maximum likelihood-based phylogenetic analyses with thousands of taxa and mixed models. Bioinformatics 22: 2688–2690, 10.1093/bioinformatics/btl446 16928733

[45] Felsenstein J (1985) Confidence limits on phylogenies: an approach using the bootstrap. Evolution 39: 783–791, 10.2307/2408678 28561359

[46] Miller MA, PfeifferW, Schwartz T (2010) Creating the CIPRES science gateway for inference of large phylogenetic trees. In: Institute of Electrical and Electronics Engineers. Proceedings of the Gateway Computing Environments Workshop (GCE) 14 Nov 2010, New Orleans: 45–52.

[47] Šutara J (2008) *Xerocomus* s.l. in the light of the present state of knowledge. Czech Mycol 60(1): 29–62.

[48] Ladurner H, Simonini G (2003) *Xerocomus* s.l. Fungi Europaei 8, Edizioni Candusso, Alassio.

[49] Watling R (2008) A manual and source book of the boletes and their Allies Synopsis Fungorum 24, Fungiflora, Oslo.

[50] Chiu WF (1948) The Boletes of Yunnan. Mycologia 40(2): 199–231.

[51] Imazeki R (1952) The Boletaceae of Japan. Nagaoa 2: 30–46.

[52] Corner EJH (1972) *Boletus* in Malaysia. Government Printing Office, Singapore.

[53] Bi ZS, Loh TC, Zheng GY (1982) Basidiomycetes from Dinghu Mountain of China I. Some new species of Boletaceae (1). Acta Bot Yunnanica 4(1): 55–64 (in Chinese).

[54] Bi ZS, Li TH, Zheng GY, Li C (1984) Basidiomycetes from Dinghu Mountain of China. III. Some species of Boletaceae (2). Acta Mycol Sin 3(4): 199–206 (in Chinese).

[55] Bi ZS, Zheng GY, Li TH, Wang YZ (1990) Macrofungus Flora of the Mountainous District of North Guangdong. Guangdong Science & Technology Press, Guangzhou (in Chinese).

[56] Bi ZS, Zheng GY, Li TH (1994) Macrofungus Flora of Guangdong Province. The Chinese University Press, Hongkong (in Chinese).

[57] Bi ZS, Li TH, Zhang WM, Song B (1997) A Preliminary Agaric Flora of Hainan Province. Guangdong Higher Education Press, Guangzhou (in Chinese).

[58] Nagasawa E (1989) Boletaceae In: Imazeki R, Hongo T (eds) Colored illustrations of mushrooms of Japan II (in Japanese). Hoikusha, Osaka: 1–44.

[59] Zang M (2006) Boletaceae (I) Flora Fungorum Sinicorum 22, Science Press, Beijing (in Chinese).

[60] Horak E (2011) Revision of Malaysian species of Boletales s.l. (Basidiomycota) described by E. J. H. Corner (1972, 1974). Malayan Forest Records 51, Kuala Lumpur.

[61] Zhang M, Huang H, Deng WQ, Shen YH, Song B (2014) The resources of boletes in Chebaling National Nature Reserve, Guangdong Province. Edible Fungi of China 33(2):10–12

[62] Singer R (1947) The Boletineae of Florida with notes on extralimital species III. Am Midl Nat 37: 1–135.

[63] Snell WH, Dick EA (1970) The boleti of northeastern North America. Cramer, Vaduz.

[64] Smith AH, Thiers HD (1971) The Boletes of Michigan. University of Michigan Press, Ann Arbor.

[65] Pegler DN, Young TWK (1981) A natural arrangement of the Boletales, with reference to spore morphology. Trans Brit Mycol Soc 76(1): 103–146, 10.1016/S0007-1536(81)80013-7

[66] Both EE (1993) The boletes of North America A compendium. Buffalo Museum of Science, Buffalo.

[67] Heinemann P, Rammeloo J (1995) Taxa nova Boletineae africanae. Bull Jard Bot Nat Belg 64(1–2): 215–216.

[68] Watling R, Li T-H (1999) Australian boletes A preliminary survey. Royal Botanic Garden Edinburgh, Edinburgh.

[69] Bessette AE, Roody WC, Bessette AR (2000) North American boletes. A color guide to the fleshy pored mushrooms. Syracuse University Press, New York.

[70] Lannoy G, Estadès A (2001) Flore Mycologique d’Europe 6—Les Bolets Documents Mycologiques, Mém. hors série 6, Lille.

[71] Muñoz JA (2005) *Boletus* s.l. (excl. *Xerocomus*) Fungi Europaei 2 Edizioni Candusso, Alassio.

[72] Ortiz-Santana B, Lodge DJ, Baroni TJ, Both EE (2007) Boletes from Belize and the Dominican Republic. Fungal Divers 27: 247–416.

